# Curcumin Hybrid Lipid Polymeric Nanoparticles: Antioxidant Activity, Immune Cellular Response, and Cytotoxicity Evaluation

**DOI:** 10.3390/biomedicines10102431

**Published:** 2022-09-29

**Authors:** María Isabel Quirós-Fallas, Krissia Wilhelm-Romero, Silvia Quesada-Mora, Gabriela Azofeifa-Cordero, Luis Felipe Vargas-Huertas, Diego Alvarado-Corella, Juan José Mora-Román, José Roberto Vega-Baudrit, Mirtha Navarro-Hoyos, Andrea Mariela Araya-Sibaja

**Affiliations:** 1 Laboratorio BIODESS, Escuela de Química, Universidad de Costa Rica, San Pedro de Montes de Oca, San José 2060, Costa Rica; 2 Laboratorio Nacional de Nanotecnología LANOTEC-CeNAT-CONARE, Pavas, San José 1174-1200, Costa Rica; 3 Departmento de Bioquímica, Escuela de Medicina, Universidad de Costa Rica, San Pedro de Montes de Oca, San José 2060, Costa Rica; 4 Facultad de Farmacia, Universidad de Costa Rica, San Pedro de Montes de Oca, San José 2060, Costa Rica; 5 Laboratorio de Investigación y Tecnología de Polímeros POLIUNA, Escuela de Química, Universidad Nacional de Costa Rica, Heredia 86-3000, Costa Rica

**Keywords:** curcumin, hybrid lipid polymeric nanoparticles, Immune cellular response, cytotoxicity, antioxidant activity, nanoparticle characterization

## Abstract

Poor solubility and short biological half-life present a challenge that needs to be overcome in order to improve the recognized bioactivities of curcumin (CUR), the main phenolic compounds derived from the roots of *Curcuma longa*. However, drug delivery systems have proven to be an excellent strategy to improve and obtain greater bioavailability. Our previous studies on curcuminoid hybrid nanoparticles have shown promising results by significantly increasing the solubility of desmethoxycurcumin (DMC) and bisdemethoxycurcumin (BDM). In this contribution, we performed a detailed characterization of a CUR as well as in vitro and in vivo studies. The developed method produced CUR loaded nanoparticles with an average size of 49.46 ± 0.80. Moreover, the FT-IR analysis confirmed the encapsulation, and TEM images showed their spherical shape. The NP achieved an encapsulation efficiency greater than 99%. Further, the release studies found that the NPs obtained a significantly higher release than the pure compounds in water. In vivo delayed-type hypersensitivity (DTH) studies showed promising results by enhancing the immune activity response of CUR in NP compared to bulk CUR. Furthermore, we report a significant increase in antioxidant activity for CUR-NP in aqueous solution compared to free CUR. Finally, an important in vitro cytotoxic effect on gastric AGS and colon SW620 adenocarcinoma cell lines was found for CUR-NP while empty carrier nanoparticles are observed to exhibit low cytotoxicity, indicating the potential of these CUR-PLU NPs for further studies to assess their phytotherapeutic applications.

## 1. Introduction

Phytochemicals are secondary metabolites that occur naturally in plants, and in recent years have gained considerable public and scientific interest due to their beneficial properties [1,2]. Curcumin is the main phenolic compound derived from the roots of *Curcuma Longa*, which has recognized bioactivities that include antioxidant, anti-inflammatory, antimicrobial, antidiabetic, and immunomodulatory activity [3,4,5].

However, curcuminoids present a poor absorption, rapid metabolism and elimination of curcumin resulting low bioavailability via the oral route [6], which is the main limitation of applicability of curcuminoids as drugs [7,8]. In recent years, nanoparticle formulation has proven to be an effective approach for protection against degradation due to food processing and digestion that occurs in the gastrointestinal tract (low pH and enzyme degradation) [9,10], increasing the pharmacokinetic routes resulting in a higher bioavailability of curcumin following oral application [11,12].

Recently, many in vitro and in vivo experiments using curcuminoids have been performed, from cell cultures to clinical trials [13,14,15,16,17]. In fact, CUR interacts with different proteins and may act by directly binding and modulating their bioactivities, or may exert indirect regulation on their functions. For instance, CUR can affect numerous molecular targets as well as modulate multiple cellular signaling pathways [18]. In addition, it is known that CUR is quite safe in humans; however, its low bioavailability constitutes a limitation for its clinical applications [1,19].

In this work, we developed hybrid lipid-polymeric nanoparticles loaded of CUR and a detailed characterization of them has been conducted through different techniques including encapsulation efficiency, Fourier transform infrared spectroscopy (FT-IR), dynamic light scattering (DLS), thermal analyses, and transmission electron microscopy (TEM). Furthermore, in vitro studies including drug release, cytotoxicity on gastric and colon adenocarcinomas cell lines, antioxidant activity through 2,2-diphenyl-1-picrylhidrazyl (DPPH) and in-vivo Delayed-type hypersensitivity (DTH) reaction have been performed.

## 2. Materials and Methods

### 2.1. Materials

Curcumin (CUR) was obtained and isolated from Costa Rican *Curcuma longa* from commercial cultivars by BIODESS Laboratory (Costa Rica). CUR analytical standard for quantification, Pluronic^®^ F-127 (PLU), cholesterol (CHO), 2,2-diphenyl-1-picrylhidrazyl (DPPH), phosphoric acid (H_3_PO_4_) and MTT (3-(4,5-dimethylthiazolyl-2)-2,5-diphenyltetrazolium bromide), were purchased from Sigma–Aldrich (Saint Louis, MO, USA) and disodium hydrogen phosphate Sodium dihydrogen phosphate monohydrate was acquired from Merck (Kenilworth, IL, USA). Polysorbatum 80 (Tween 80) was obtained from Sonntag & Rote S.A. (San José, Costa Rica), and Sorbitan monooleate (Span^®^ 80) was supplied by LABQUIMAR S.A (San José, Costa Rica). Organic solvents chloroform (CHCl_3_), methanol (MeOH), and acetonitrile (MeCN) were purchased from JTBaker (Phillipsburg, NJ, USA), and were all HPLC/UV grade or highly pure, and water was purified using a Millipore system filtered through a Millipak 40 (Millipore membrane 0.22 µm). Finally, the human gastric adenocarcinoma cell line AGS, human colorectal adenocarcinoma SW 620 were obtained from the American Type Culture Collection (ATCC, Rockville, MD, USA).

### 2.2. Preparation of CUR Loaded Nanoparticles (CUR-NP)

CUR nanoformulation was prepared according to Wilhelm et al. (2021) [8]. The method consisted of preparing an emulsion adding the aqueous phase into the organic phase one and applying high-speed homogenization. The organic phase was composed of 5 mg of CUR and 120 mg of CHO dissolved in 6 mL of MeOH: CHCl_3_ 1:1 solvent mixture. The aqueous phase was prepared by dissolving 250 mg of PLU in 50 mL of acetic acid 0.1% and adding 2 mL of a 1:1 mixture of Tween 80:Span80. The aqueous phase addition speed was approximately 3 mL/min and mixed at 16,000 rpm for 10 min using an IKA ULTRA-TURRAX^®^ T25 high speed homogenizer. The nanoparticles were collected and washed three times with ultrapure water by ultracentrifugation using a Thermo Scientific Sorvall ST 16R centrifuge at 12,000 rpm for 40 min at 10 °C each cycle in order to remove unencapsulated CUR or unreacted starting materials and, filtered through an ADVANTEC^®^ ultrafilter unit. The final formulation was refrigerated after dispersion in 5 mL of purified water containing 0.01% Tween80^®^ for in vitro evaluation and lyophilized for further physicochemical characterization. A blank of the nanoparticles (Plain-NP) was prepared following the previously method without adding CUR in the organic phase.

### 2.3. Physicochemical Characterization of CUR-NP

#### 2.3.1. Fourier Transform Infrared Spectroscopy (FT-IR)

FT-IR spectra were collected in the range of 4000–600 cm^−1^, at 32 scans from a Thermo Scientific Nicolet 6700 spectroscope equipped with a diamond attenuated total reflectance (ATR) accessory at a resolution of 4 cm^−1^. The samples were measured without any dilution by placing them directly into the ATR cell.

#### 2.3.2. High Resolution Transmission Electron Microscopy (HR-TEM)

HR-TEM images were acquired using a JEOL, JEM2011 HR-TEM at an acceleration voltage of 120 kV. 5 uL of the samples were placed on a sample holder grid and dried under a nitrogen atmosphere.

#### 2.3.3. Dynamic Light Scattering (DLS)

Particle size (PS, z-average) and polydispersity index (PI) were determined at 25 °C using a Malvern Nano Zetasizer ZS90 instrument using medium refractive index 1.33, and viscosity of 0.8872 cP under 90°. To achieve appropriate concentrations, the samples were diluted with deionized water.

#### 2.3.4. Encapsulation Efficiency (%EE)

The %EE was determined using the direct method quantifying the real amount of CUR into the nanoparticle by extracting the metabolite out of the formulation. To achieve this goal, 100 µL of fresh samples and 900 µL of MeOH were mixed. The solution was filtered through a polyamide membrane of 0.45-μm pore size placed in a Sartorius stainless steel syringe filter holder. The solutions were analyzed in a Dionex Ultimate 3000 UHPLC system provided with variable wavelength detector, pump, variable temperature compartment column and autosampler. The elution was performed in a Nucleosil 100-5 C18 column (250 mm × 4.0 mm, 5 μm) at a temperature of 35 °C using 55% of MeCN and 45% of H_3_PO_4_ 0.1% as mobile phase at a flow rate of 1 mL/min and setting down the detection at 420 nm. The EE percentage for each formulation was calculated through the Equation (1).

(1) $$\%EE_{D}=\frac{Drug\;in\;nanoparticle\;}{Total\;drug\;added}\times 100$$

#### 2.3.5. Differential Scanning Calorimetry (DSC)

DSC curves of the samples were obtained from a DSC-Q200 calorimeter provided with a TA Refrigerated Cooling System 90, both TA Instruments branded. Samples weighted in the range of 2 to 5 mg were placed into unsealed aluminum pans with lids. The samples were analyzed from 40 to 250 °C set up with a heating rate of 10 °C/min and under a dynamic nitrogen atmosphere of 50 mL/min.

### 2.4. In Vitro Studies of CUR-NP

#### 2.4.1. Release Profile of CUR-NP

The in vitro release profile of CUR-NP formulation was estimated using two different dissolution media. The first one corresponded to water (M-1) and the second was phosphate buffered saline of pH 7.4 containing MeOH 20% and 2.5% of Tween 80 (M-2). 1 mL of each formulation was placed in 80 mL of the respective medium maintained at 37 ± 0.5 °C and 150 rpm constant agitation in a Labnet 211 DS shaking incubator. A volume of 4 mL of each solution were withdrawn at specific time intervals without replacing the volume. The aliquots were centrifuged at 6000 rpm for 10 min in a Thermo Scientific Sorvall ST 16R centrifuge maintaining at 37 °C. The concentration of CUR was determined using a spectrophotometer Shimadzu 1800 double beam UV-Vis at a wavelength of 420 nm. Dissolution profiles of free CUR was evaluated in both dissolution media for comparison with CUR release from the developed NP. The sampling was done in triplicate.

#### 2.4.2. DPPH Radical-Scavenging Activity

DPPH evaluation was performed as previously reported [20], for free and nanoencapsulated CUR. Free CUR samples were evaluated in ethanolic and aqueous solution. CUR nanoparticles samples were prepared in water. Briefly, a solution of 2,2-diphenyl-1-picrylhidrazyl (DPPH) (0.25 mM) was prepared using ethanol as solvent. Next, 0.5 mL of this solution were mixed with 1 mL of the respective free CUR or CUR-NP solution at different concentrations and incubated at 25 °C in the dark for 30 min. Solutions of Trolox in both solvents, water, and ethanol were used as standards [21]. Blanks were prepared for each sample and DPPH absorbance was measured at 517 nm. The inhibition percentage was determined as shown in Equation (2)

(2) $$Inhibition\;percentage\;\left(\%\right)=\frac{\left(Abs_{blank}-Abs_{sample}\right)}{Abs_{blank}}\times 100$$

The percentage of the radical-scavenging activity of the sample and Trolox was plotted against its concentration to calculate IC_50_, which corresponds to the amount of sample necessary to reach the 50% radical-scavenging activity. Each sample was analyzed in three independent assays.

#### 2.4.3. Evaluation of the Cellular Immune Response of Mice through DTH

Animals reared in the Biological Testing Laboratory (LEBi, for its Spanish acronym) of the Universidad de Costa Rica were used. The assay was performed at the bioterium of the Faculty of Pharmacy (conditions: constant temperature of 22 to 24 °C, relative humidity of 60 to 70%, and light and dark cycles of 12 h each). The animals had access to water and food ad libitum. Their handling and care were approved by CICUA-023-2020 permit from the Institutional Committee for the Care and Use of Animals (CICUA, for its Spanish Acronym) at session No. 200-2020.

Female mice of the C3H/He strain aged two to four-month-old were divided into different groups of three animals and each group was put in a separate cage. Afterwards, groups 1 and 2 were orally administered water and groups 3 and 4 were orally administered aqueous CUR or CUR-NP in a dose of 40 mg/Kg per animal respectively, for five weeks, from Monday to Friday, thus for 36 days. Animals in groups 2 to 4 were immunized with Fissurella latimarginata hemocyanin (FLH) antigen (50 µg) subcutaneously in their right footpad on days 7 and 21, while animals in Group 1 were used as control without the application of FLH.

The delayed-type hypersensitivity (DTH) evaluation followed former studies [22]. The right and left foot pad thickness of each animal was measured with a Krœplin C1R10 (Krœplin GmbH), at day 39 before the injection of 50 µg of FLH (day 39) and also at days 40, 41 and 42, corresponding to 24, 48 and 72 h after FLH injection respectively.

#### 2.4.4. Evaluation of Cytotoxicity on Tumoral Cells

##### Cell Culture

Human colorectal adenocarcinoma SW 620 cell lines and human gastric adenocarcinoma AGS cell lines were grown in minimum essential Eagle’s medium (MEM) with 10% fetal bovine serum (FBS), 100 IU·mL^−1^ penicillin, 0.25 μg/mL amphotericin B, 2 mmol/L glutamine and 100 μg/mL streptomycin. The conditions used for cell growth comprised a humidified atmosphere with 5% CO_2_ at 37 °C and these cell lines were sub-cultured by using trypsin–EDTA solution at 70–80% confluence for detaching. Afterwards, a volume of 100 μL of cell suspension (1.5 × 105 cells/mL) was seeded in 96-well plates overnight. These cells were exposed to different concentrations of either CUR-NP (50 μL), or Plain-NP (50 μL), diluted with cell culture medium to final concentrations between 15–500 μg/mL, for a total of 48 h. Control cultures were prepared without the addition of any formulation to culture medium.

##### Assessment of Cytotoxicity by MTT Assay

MTT assays were performed in order to assess the cell viability after the 48h incubation period. The cytotoxic activity of the samples correlates to a decrease in the cell line viability. To perform the assay, the medium was eliminated, and the cells were washed twice using 100 μL of Phosphate Buffer Saline (PBS). Subsequently, the cells were incubated with 100 μL of MTT (5 mg/mL) for 2 h at 37 °C. Afterwards, the formazan crystals formed were treated with 100 μL of ethanol 95% for dissolution. Finally, the absorbance was measured in a microplate reader at 570 nm. For each sample and concentration, dose–response curves were established and the IC50 value (cell viability reduced by 50%) was calculated.

### 2.5. Statistical Analysis

To determine differences in DPPH antioxidant activity and in DTH values in respect to the footpad thickness, one-way analysis of variance (ANOVA) with a Tukey post hoc as statistical tests were applied.

## 3. Results

### 3.1. Encapsulation

**Figure 1:**
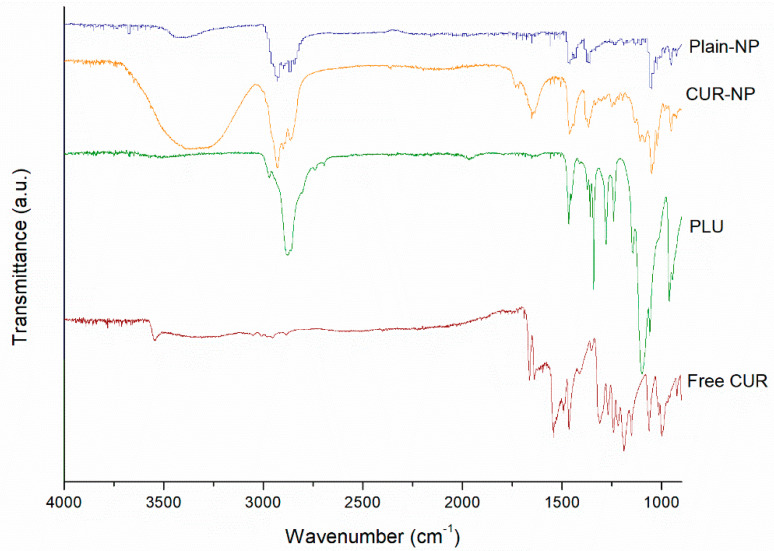
FT-IR spectra of CUR loaded PLU-NP, Plain-NP, and free CUR.

FT-IR spectral analysis clarifies any possible chemical interaction through the probable bonding between drug and core components during the preparation process [21]. The FT-IR spectra of the components of nanoparticles, Plain-NP, free CUR, as well as the curcuminoid loaded NP were evaluated between 4000 and 1000 cm^−1^ and the results are shown in Figure 1. The major signals of CUR in the spectra were similar to those reported in earlier studies [8,23].

The FT-IR spectra of the CUR-NP revealed the distinctive absorption peaks of CUR, indicating a mixture of signals from the curcuminoid, PLU, and CHO components. For instance, signals at 1465 cm^−1^ and 1046 cm^−1^ correspond to CH_2_ and CH_3_ deformation vibrations, ring deformation from CHO [24]. In addition, the main characteristic signals of CUR in the FT-IR spectra of CUR-NP correspond to 3339 cm^−1^ was related to the stretching vibration of hydrogen-bonded -OH, 2937 cm^−1^ was assigned to the stretching vibrations of methyl (CH_3_), to 2857 cm^−1^ was associate to C-H stretch aliphatic, 1648 cm^−1^ was assigned to C-H-C stretching vibration, 1352 cm^−1^ correspond to in-plane O-H bend and 1055 cm^−1^ was related to C-O stretch, these signals for the CUR in the hybrid NPs further demonstrating that the drug was loaded onto the nanoparticle core. The PLU signals in the FT-IR spectra of CUR-NP and the Plain-NP correspond to 2896 cm^−1^ was associated to C-H stretch aliphatic, 1369 cm^−1^ was assigned to in-plane O-H bend, 1045 was related to C-O stretching of aliphatic ether group [25]. These signals provide information about the interaction between the polymer and the drug, if these compounds interact, then would be band shifts and broadening in the functional groups in the FT-IR spectra compared to the spectra of the free CUR and polymer [26]. The spectral analysis of Plain-NP and CUR-NP exhibited peaks which were a summation of the characteristic peaks obtained with the free CUR and pure components, indicating no interaction between the structural components of the system and CUR molecules. This showed that there was no chemical interaction of the drug with carriers.

### 3.2. Encapsulation Efficiency (%EE)

Inner lipid cores can incorporate lipophilic compounds such as curcuminoids efficiently in their bilayers, the external core act as protector to enhance their cellular uptake while protecting the drug from photo and chemical degradation [27]. The percentage entrapment or encapsulation efficiency (%EE) determines the amount of drug that has entered the lipid inner core, and it depends on combinatorial factors. The CUR-NP formulation exhibited an 99% of efficiently loaded CUR into de nanoparticle, in agreement with the previous studies for DMC and BMC hybrid nanoparticles [8]. This high %EE value can be related to the strong interaction between the phenyl groups on CUR structure loaded into inner core with the lipid. Thus, PLU provides the forces to assembly via the attraction between alkyl groups in the polymer and aromatic groups [28]. Because of this amphiphilic nature, the external core of the nanoparticle will self-aggregate in aqueous solutions to form spherical micelles with hydrophobic PEO–PPO–PEO coronas.

### 3.3. Particle Size and Morphology

The mean values of size and size distribution are physicochemical parameters that must be modulated for the optimal development of a formulation. The average particle size and the polydispersity index (PDI) of CUR-NP are present in Table 1. The minimum size that can be achieved depends on the viscosity of the materials and homogenization parameters, in our previous investigation it was determined that a high speed of homogenization (<16,000 rpm) generated smaller particle sizes [8]. When applying these parameters to the CUR-NP, it was found that the particle size was <50 nm. Therefore, the PDI is a dimensionless measure of the breadth of the particle size distribution [29,30]. Hence, as previously reported in the literature a PDI value should be < 0.3 to indicate monodisperse behavior in the sample [31,32]. The PDI of CUR formulation lies between 0 and 0.3 which indicated that the particle size exhibited narrow distribution and displayed suitable nanoparticle formulation [33].

**Figure 2:**
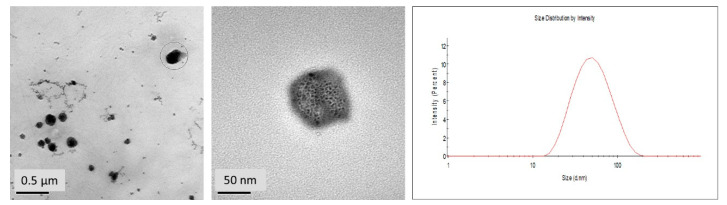
HR-TEM images and size distribution histogram of CUR-NP.

Furthermore, micrographs of TEM (Figure 2) revealed that the CUR-NP were smooth spherical shaped and highly monodispersed with an average diameter of 50 nm, consistent with dynamic light scattering measurements. The nanoparticles showcased a lighter outer layer, i.e., the surrounded lipid membrane and a darker inner CUR core, as previously reported by Akhlaghi in 2019 [34].

### 3.4. Thermal Analysis

**Figure 3:**
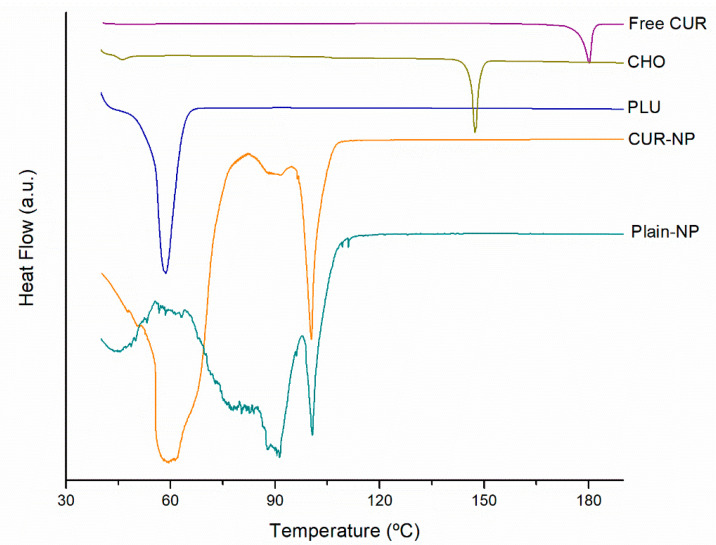
DSC curves of PLHN main components and the CUR loaded formulation.

Differential Scanning Calorimetry (DSC) thermograms of Plain-NP, CUR-NP, free CUR, and PLU are presented in Figure 3. Free CUR display melting temperatures at 184 °C [35]. The CUR-NP did present a sighlyty amorphous peak at 60 °C corresponding to the melting temperature of PLU. Futhermore, CHO present a peak at 147 °C, corresponding to monohydrate form melting temperature [36] Plain-NP was characterized by the existence of a endothermic peak at 119.90 °C, meanwhile CUR-NP showed a sharp peak at 125.76 °C, this event can be associated with polymorphic forms and phase transitions reported for CHO [37] that could be occurring during NP preparation. This implies from DSC data that the drug is present in crystalline form in the nanoformulation, but the crystallinity has been reduced as compared to the pure drug. This absence of the characteristic endothermic peak of CUR implies that the curcuminoids were dispersed within the nanoparticle matrix [38,39]. It is usually preferred that the drug in the nanoformulation is amorphous, resulting in better absorption and bioavailability [40]. Additionally, these observations were in agreement with the statement of no evidence of incompatibilities between CUR and formulation constituents.

**Figure 4:**
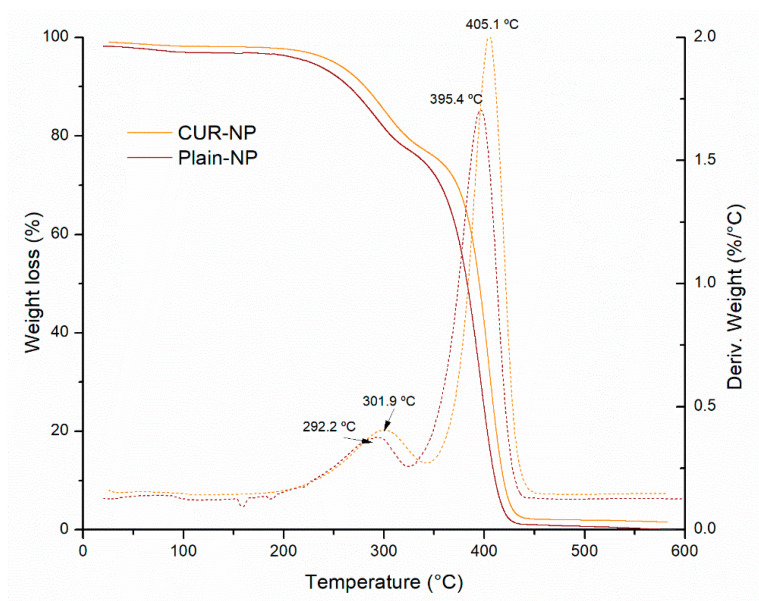
TGA curves of Plain-NP and CUR-NP. Dashed and dotted lines represent the derivative of % weight loss as a function of temperature.

Figure 4 shows the thermogravimetric (TGA) curves of the Plain-NP and CUR-NP. Similar thermal behavior was observed for both hybrid nanoparticles. We observed three mass loss events at 81.9 °C that can be associated with water loss, at around 300 °C and 400 °C. Only a slight difference in the thermal decomposition of CUR-NP was observed starting its weight loss at a slightly lower temperature than the Plain NP. According to Hefferman et al. (2017), free CUR showed a weight loss at around 184 °C [41]. Different researchers reported that PLU demonstrate higher initial decomposition temperature but decomposed immediately, the copolymer PLU occurred between 300 °C to 400 °C [42].

### 3.5. In Vitro Release Evaluation

**Figure 5:**
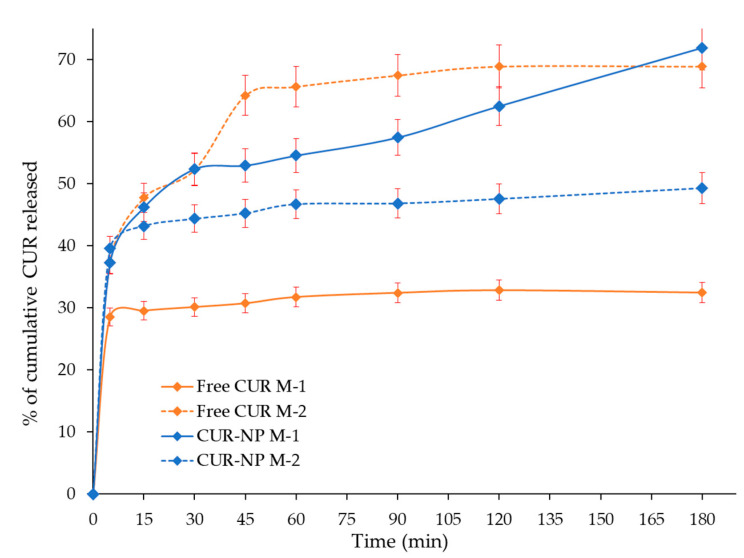
Release profile of CUR from the NP compared to free CUR dissolution rate in two dissolution media. Error bars represent the standard deviation of CUR concentration in the triplicates.

In the in vitro release evaluation of CUR from the nanoparticle, several dissolution media and pH values were tested with anomalous results attributed to the chemical instability of curcuminoids at other pHs different from 7–8. The dissolution media test included sodium lauryl sulphate 0.25% (pH 8.5) which is considered a biorelevant medium. However, there were inconveniences with the formulation since it precipitated affecting the timely release of the nanoparticles as reported by Abouelmagd et al. (2015) [43] and Baby et al. (2021) [44]. Chemical instability of CUR during release tests have been controlled using delivery media with pH conditions in the lumen of the intestine with a range of 6.8-7.4 (colon pH ~7.4) [45,46,47]. Therefore, the in vitro release profiles of CUR from the hybrid nanoparticles CUR-NP and the Plain-NP were evaluated in two different dissolution media M-1 (water, pH 6.8) and M-2 (phosphate buffered containing MeOH 20% and 2.5% of Tween 80, pH 7.4) over the period of 120 min (Figure 5). According to Gupta et al. (2020), the release tests were conducted within the half-life time range of CUR and there was no evidence of chemical degradation [48].

Even though the use of water is not recommended as a release medium for poorly water-soluble molecules, it was tested to evaluate the aqueous solubility enhancement exerted by the synthesized nanosystems. In addition, because the interest on evaluation the effect of Pluronic backbone degradation in water that releases the loaded compounds that has been associated with water solubility enhancement [49,50,51].

The release profile of CUR-NP in M-1 (pH 7.4) appeared to release a higher amount of CUR compared to that in M-2 (water, pH 6.8). Nevertheless, comparing dissolution profile of free CUR with curcumin release from PLU NP in M-1 it is observed that CUR-NP obtain a release of 73% at 180 min compared with free CUR, which associate a release of 32% at 180 min. In turn, when comparing dissolution profile of free CUR with CUR release from NP in M-2, it is observed that CUR-NP have a control release compared to free CUR, which presents a burst of 70% at 40 min. At 180 min, the release of the hybrid nanosystem was around 51% while free CUR does not show a significant increase after the burst. A possible explanation for this may be due to the presence of MeOH suggesting an increased burst dissolution rate of free curcuminoid and higher, but controlled release rate of curcuminoids from the nanoparticle.

It is interesting to note that the dissolution profile of CUR-NP is higher in water; this increased release from the hybrid nanoparticles can be attributed to the interaction between CUR and the bilayer, as the hydrophobic interaction between them becomes weak, the shell core of PLU will break, exhibiting fast and sustained release of the molecule [52]. The large surface area, a high diffusion coefficient due to small molecular size, low viscosity in the matrix and a short diffusion distance δ for the drug (i.e., release from the outer surface region of the nanoparticle) [49,50,51] are other factors that could contribute to an efficient release.

### 3.6. Antioxidant Activity Evaluation of the Free CUR and CUR-NP

Antioxidant activity of free and nanoencapsulated CUR was studied through DPPH analysis, using Trolox as the reference compound, as described in the materials and methods (Section 2.4.2). Results of the antioxidant activity are shown in Table 2.

As observed, DPPH values are similar for Trolox when using ethanol or water as solvents and a One-way ANOVA with a Tukey post hoc as statistical test showed non-significant difference (*p* < 0.05) between those values, therefore indicating the DPPH test delivered adequate results in both media. Meanwhile, results for free CUR in ethanol and water were significantly different with free CUR in ethanol, presenting a low IC_50_, indicating important antioxidant activity while free CUR in aqueous solution showed a much higher IC_50_, which aligns with CUR’s low solubility in water [53]. In turn, DPPH assay of CUR nanoparticles in aqueous solution showed a low IC_50_ hence a much higher antioxidant activity than free CUR in aqueous solution.

In fact, One-way ANOVA analysis indicated IC_50_ of CUR nanoparticles was shown to have no significant difference (*p* < 0.05) to that of free CUR in ethanolic solution, evidencing that the antioxidant activity was significantly enhanced by nanoencapsulation. These observations are consistent with previous results on nanoparticle formulations of curcuminoid mixtures [54] and individuals CUR [55,56], DMC and BDM [8].

### 3.7. Cytotoxic Activity Evaluation of Free CUR and CUR-NP

CUR has reported therapeutic value in terms of its anticancer potential but the main obstacle faced is its low aqueous solubility and consequently in bioavailability [57]. Some reports have evaluated free CUR in aqueous solution and no cytotoxicity was observed in tumoral cell lines [58,59]. For this reason, recent studies have assessed nanoparticles improving the solubility of CUR as an alternative to deliver CUR to carcinoma cells [57,60].

**Figure 6:**
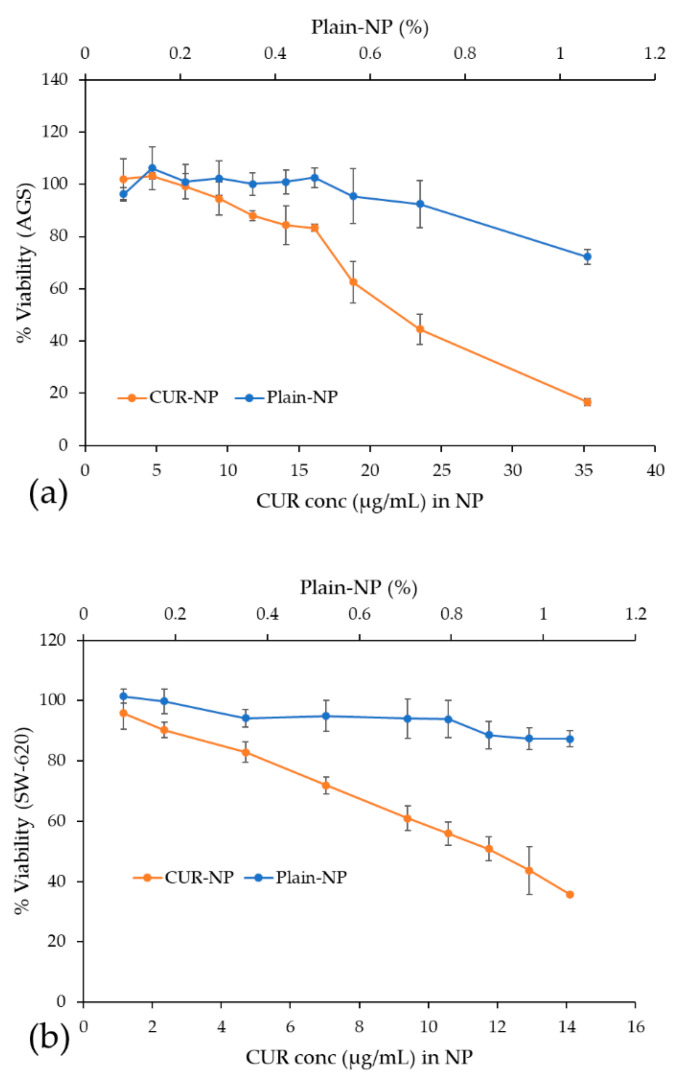
Cytotoxicity dose–response curves of CUR-NP and Plain-NP in AGS and SW620 tumor cell lines. Results are presented as mean ± SD of three independent experiments. (**a**) Samples in AGS cells (**b**) Samples in SW620 cells.

As described earlier, the CUR-NP formulation in this study was able to disperse in an aqueous media, which is used in our cell culture media in the cytotoxicity assay, avoiding toxic compounds as MeOH or DMSO, traditionally required to dissolved CUR. As presented in Figure 6, the nanoencapsulated CUR achieved a dose-dependent cytotoxic effect against gastric (AGS) and colon (SW-620) adenocarcinoma cells, after 48 h. For AGS cells, the 50% cytotoxic activity was achieved with 21.6 ± 0.8 µg/mL (58.6 ± 2.1 µM) and for SW-620 cells with 12.5 ± 0.4 µg/mL (33.9 ± 1.0 µM).

Compared with reports from the literature, our results for CUR-Fe_3_O_4_ cellulose nanocrystals stabilized with Pickering emulsion did not achieve a 50% of cytotoxicity with 30 µg/mL even after 96 h against colorectal carcinoma cells (HCT-116) [61]. On the other hand, a better cytotoxic activity was observed in a different colon adenocarcinoma cell (HT-29) by CUR-chitosan-nanoparticles showing an IC_50_ of 10.2 µM [57] and 20 µM [62] after 48 h treatment. On gastric tumoral cell lines, co-delivery of etoposide and curcumin by lipid nanoparticles showed promising cytotoxicity results with an IC_50_ of 2 µM on SGC7901 human gastric adenocarcinoma cells [63]. However, the comparison of cytotoxic effects between these studies is complex because of the diverse composition of the nanoparticles and the particular characteristics of the tumoral cell lines.

Finally, in Figure 6, it can also be observed that for both adenocarcinoma cell lines, AGS and SW-620, in contrast to the nanoencapsulated CUR, the viability of cells incubated with Plain-NP remained at about 80% relative to untreated cells within the assessed concentration. This indicates that Plain-NP had negligible toxicity to cells and confirms its biocompatibility.

### 3.8. Evaluation of the Cellular Immune Response of Mice through DTH

**Figure 7:**
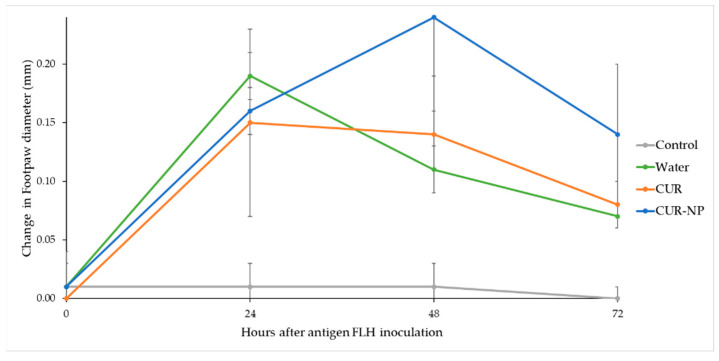
Delayed-type hypersensitivity (DTH) test expressed as change in footpad thickness for each treatment measured at 0, 24, 48 and 72 h.

The delayed-type hypersensitivity (DTH) reaction in immune response is a technically simple test capable of reflect the development of systemic antigen-specific immunity when exposing the subject to different treatments. The data presented here demonstrates that antigen-specific DTH responses are influenced by the daily consumption of CUR and its nano-formulation, with this one being the sample with the greatest responsiveness at 24 and 48 h, as shown in Figure 7.

As shown in Table 3, the samples showed in the one-way ANOVA different significant response at 48 h (*p* < 0.05) and CUR-NP was the one with the best immunomodulation capacity and highest enhancement of the cellular response. In fact, the mice from group 4 with the CUR-NP treatment showed increased difference, exhibiting the highest influence and effectiveness on the immune response. At 24 h, the immune response did not exhibit significant difference (*p* < 0.05) among the treated mice.

It is clearly shown that variability of the immune response from the animals when administering bulk CUR in their diets, after sensitized by FLH antigen, is lower than CUR-NP and, furthermore, the variability is not different from the animals without any previous treatment.

The DTH constituted the first experimental evidence of how immunity was transferred and carried only by the immune cells. Since this reaction is characterized by the influx of immune cells at the site of injection, the immune response was initiated by these cells [64]. Previous studies have reported tumor antigen-specific DTH responses that correlate to a determinate antigen-specific peripheral blood T-cell response [65,66]. For these properties, there is the potential to provide insight into the cellular immunomodulatory properties of the different treatment.

CUR is a potent immunomodulatory agent that has biochemical pleiotropic effects due to its numerous targets, including the regulation of cytokines and inflammatory mediators such as IL-1, IL-2, IL-6, IL-8 and IFN-γ [67]. Despite these well-known properties, the biggest gap when implementing curcumin-based therapies within the clinic field is its low bioavailability. Most preclinical trials have explored the impacts of CUR at dosages that cannot be obtained solely by oral intake. Innovatory approaches have been created to progress the bioavailability of CUR as the hybrid nanoformulation prepared and reported in this study using CHO and PLU.

In our case, it was demonstrated that when administering the CUR-NP orally, it was sufficient to positively modulate the immune response. Finally, since DTH is not an individual phenomenon, but rather a group of related immune responses to antigen exposure where innate immune is strictly necessary for mounting a T-cell-mediated delayed-type hypersentitivity reaction [68,69], these results can be considered as an indicator of the immunomodulatory capacity of the present CUR nanoformulation.

Other studies have shown that nanoformulations of CUR have immune-enhancing activity in mice while free CUR samples’ immunosuppressive potential is dose dependent, as measured by DTH test at 24 h [70]. Compared to our results at 24 h, one can assert that the oedema was higher for the animals without any CUR treatment, but when reaching the 48 h the enhanced immune activity of our nanoformulation completely changed the immune response in contrast with the water and bulk CUR treatment. Other species of *Curcuma* genus have shown similar immunomodulatory effects when testing extracts of *C. amada* and *C. mangga* [71,72].

Therefore, the upgrade of immune responses by enhancers such as CUR, when these are nano formulated, is more than desirable, since different factors from genetic traits or even aging and senescence impact in the immune system to reduce its efficacy, increasing the probabilities of the spread of cancerous malignancies or resistant infections.

## 4. Conclusions

This paper reports the successful synthesis of CUR-NP. The NPs, composed of PLU and CHO, were prepared by high-speed homogenizer method and characterized by TEM, FT-IR, TGA, DSC, to improve the therapeutic efficacy of the CUR. Further, the prepared CUR-NP exhibited a small particle size, showed monodisperse with a spherical shape, and had high encapsulation efficiency. Therefore, they were able to contribute to enhancing the solubility and stability, and manage to successfully encapsulate the curcuminoids in the hybrid nanosystems. The nanoparticle was released in a sustained manner in M-1. The DPPH radical scavenging activity indicated improvements in antioxidant activity, and showed the highest cytotoxicity in vitro. Therefore, the CUR-NP exhibited considerable improvements in vivo, and a DTH test shows that hybrid nanoparticles can improve their effects, resulting in increased antigen specific responses. In sum, these CUR nanoformulation in-vivo DTH results, along with a significant increase in DPPH antioxidant activity in aqueous solution compared to free CUR as well as an important in-vitro cytotoxic effect on gastric AGS and colon SW620 adenocarcinoma cell lines while showing that Plain-NP exhibit low cytotoxicity, indicate the potential of these CUR-NPs. Further studies are required to assess their phytotherapeutical applications.

## Figures and Tables

**Table 1:** Physical characteristics of CUR-NP.

Formulation	Size Average (nm)	Polydispersity Index (PDI)
CUR-NP	49.5 ± 0.8	0.27 ± 0.03

**Table 2:** Antioxidant activity of free CUR, CUR-NP and Trolox.

	IC_50_ (µg/mL) ^1,2,3,4^	
	Ethanol	Water
CUR	9.60 ^a,&^ ± 0.12	2444.80 ^b,^^ ± 19.68
CUR-NP ^5^	--	9.55 ^#^ ± 0.18
Trolox	7.77 ^a,≠^ ± 0.10	7.82 ^a,#^ ± 0.34

^1^ IC_50_ µg/mL. ^2^ Values expressed as mean ± standard deviation (S.D.). ^3^ Nanoparticles samples were prepared in water ^4^ Different superscript letters in the same row indicate differences are significant at *p* < 0.05 using one-way analysis of variance (ANOVA) with a Tukey post hoc as statistical test. ^5^ Different superscript signs in the same column indicate differences are significant at *p* < 0.05 using one-way ANOVA with a Tukey post hoc as statistical test.

**Table 3:** Delayed-type hypersensitivy (DTH) test expressed as change in footpad thickness for each treatment measured at 0, 24, 48 and 72 h.^1,2^.

Formulation	0 h (mm)	24 h (mm)	48 h (mm)	72 h (mm)
Control	0.01 ± 0.03 ^a^	0.01 ± 0.02 ^b^	0.01 ± 0.02 ^c^	0.00 ± 0.01 ^c^
Water (FLH)	0.01 ± 0.03 ^a^	0.19 ± 0.05 ^a^	0.14 ± 0.04 ^b^	0.07 ± 0.02 ^b^
CUR	0.00 ± 0.01 ^a^	0.15 ± 0.08 ^a^	0.14 ± 0.05 ^b^	0.08 ± 0.02 ^b^
CUR-NP	0.01 ± 0.02 ^a^	0.16 ± 0.02 ^a^	0.24 ± 0.10 ^a^	0.14 ± 0.06 ^a^

^1^ Values are expressed as mean ± Standard Deviation. ^2^ Different superscript letters in the same column indicate differences are significant at *p* < 0.05 using one-way analysis of variance (ANOVA) with a Tukey post hoc.

## Data Availability

Not applicable.
